# Interaction of Lysozyme with Poly(L-lysine)/Hyaluronic Acid Multilayers: An ATR-FTIR Study

**DOI:** 10.3390/polym15041036

**Published:** 2023-02-19

**Authors:** Natalia Velk, Janos Keller, Claus Duschl, Gerald Brezesinski, Dmitry Volodkin

**Affiliations:** 1 Fraunhofer Institute for Cell Therapy and Immunology, Branch Bioanalytics and Bioprocesses (Fraunhofer IZI-BB), Am Mühlenberg 13, 14476 Potsdam-Golm, Germany; 2 Max Planck Institute of Colloids and Interfaces, Am Mühlenberg 1, 14476 Potsdam-Golm, Germany; 3 Department of Chemistry and Forensics, School of Science and Technology, Nottingham Trent University, Clifton Lane, Nottingham NG11 8NS, UK

**Keywords:** layer-by-layer, protein, secondary structure, orientation, attenuated total reflection Fourier transform infrared spectroscopy

## Abstract

Polyelectrolyte multilayers (PEM) loaded with bioactive molecules such as proteins serve as excellent mimics of an extracellular matrix and may find applications in fields such as biomedicine and cell biology. A question which is crucial to the successful employment of PEMs is whether conformation and bioactivity of the loaded proteins is preserved. In this work, the polarized attenuated total reflection Fourier transform infrared (ATR-FTIR) technique is applied to investigate the conformation of the protein lysozyme (Lys) loaded into the poly(L-lysine)/hyaluronic acid (PLL/HA) multilayers. Spectra are taken from the protein in the PEMs coated onto an ATR crystal during protein adsorption and desorption. For comparison, a similar investigation is performed for the case of Lys in contact with the uncoated crystal. The study highlights the presence of both “tightly” and “poorly bound” Lys fractions in the PEM. These fractions differ in their conformation and release behavior from the PEM upon washing. Comparison of spectra recorded with different polarizations suggests preferential orientation of alpha helical structures, beta sheets and turns in the “tightly bound” Lys. In contrast, the “poorly bound” fraction shows isotropic orientation and its conformation is well preserved.

## 1. Introduction

Polyelectrolyte multilayers (PEMs) loaded with bioactive compounds serve as an attractive candidate for use as substrates for drug delivery, tissue engineering and other biotechnological fields [1,2]. One of the central questions for applications of PEMs in the biosciences is whether the secondary structure of biomolecules loaded into the films is retained. Attenuated total reflection Fourier transform infrared (ATR-FTIR) spectroscopy is a powerful technique used to address this issue.

The possibility of coating the ATR crystal with a film makes the ATR-FTIR technique widely applicable to the analysis of polymer conformation and the structure of polymer systems such as PEMs [3]. In spite of the versatility of the ATR-FTIR technique, to date, only a few studies have been devoted to the investigation of the internal interactions in “pure” PEMs as well as PEMs with adsorbed, embedded or loaded proteins. Important quantitative information on the compensation of charges in the interior of poly(L-lysine)/hyaluronic acid (PLL/HA) multilayers has been gained by Crouzier and Picart, 2009 [4] using this technique. A number of works provided indications that the secondary structure of proteins adsorbed onto/into various kinds of PEMs is retained [5,6,7].

The present study deals with the investigation of the interactions between the PEMs and the biomolecule lysozyme (Lys) as a model system using polarized ATR-FTIR spectroscopy. Specifically, absorption spectra of Lys in PLL/HA multilayers are analyzed upon Lys loading into and release from the PEM. For comparison, spectra of Lys during adsorption and desorption onto/from the bare (blank) ATR crystal have been taken. Based on these spectra, a reference spectrum of Lys in solution has been obtained, which is important for precise evaluation of the secondary structure of Lys in the PEMs. Lys serves as a model molecule due to its availability, stability and well-characterized structural and spectral properties.

An approach widely used for the determination of the secondary structure of proteins is the calculation of the fractional distribution of secondary structural elements from the spectra. To achieve this, different methods have been developed [8,9,10,11,12]. One can perform a comparison of the parameters obtained from spectra recorded from the sample (here, Lys found in the PEMs or adsorbed on the bare crystal) and reference spectrum (here, Lys in solution) with literature data. Based on this, it is possible to evaluate whether and to what extent the native protein structure is affected; for instance, by the specific environment (here, the PEM). However, such comparison is prone to misinterpretation, as quantitative calculation of secondary structure elements can be sensitive to the water content in the spectra and to the choice of parameters needed for the calculation [13]. That is why, in the present study, two alternative approaches are combined. Firstly, the resemblance of the spectra recorded from Lys on the PEM-coated or blank crystals with the spectrum of the native Lys in solution is analyzed based on area overlap values, as suggested by van de Weert et al., 2001 [14]. Secondly, the approach of difference spectroscopy [15] is applied. This allows one to highlight differences between the spectra under comparison and assign them to specific secondary structure elements. Specifically, in the present study, difference spectroscopy is applied to monitor changes in the sample spectra over time. In addition, spectra recorded with different polarizations are compared for the analysis of the possible presence of specific orientations of the secondary structure elements.

## 2. Materials and Methods

*Equipment.* An FTIR-spectrometer Vertex 70 (Bruker, Germany) equipped with a lift-model single-beam-sample-reference (SBSR) ATR mirror attachment with a hydrodynamically optimized and thermostated cuvette (flow-through cell) from Optispec, (Neerach, Switzerland) has been used. A trapezoidal Germanium-crystal of 50 × 20 × 2 mm has been used as multiple internal reflection element (MIRE), resulting in 24 internal reflections at an incoming angle of 45 degrees. With parallelism < 1 arcmin and optically polished to a flatness of 1 λ at 633 nm and a S/D (scratch and dig) of 60/40, the single crystal was newly obtained from KOMPLAS Optische Komponenten und Lasersysteme GmbH, Germany. It had a cubic crystal class and a refractive index of 4.01 (at 2000 cm^−1^). A liquid nitrogen cooled mercury cadmium telluride detector collected the IR-beam. For measurements and partly for evaluation (see *Evaluation of the infrared spectra* below), the Software OPUS 6.0 (Bruker, Germany) has been used [16]. To duct the solutions (protein and buffer for adsorption and desorption studies, respectively) through the flow-through cell, a peristaltic pump (Ismatec SA, Switzerland) with a flow rate of 0.27 mL/min and Viton^®^-tubes with an inner diameter of 1 mm were used. Viton O-rings were used for sealing.

*Design of the flow-through cell and recording of spectra.* The SBSR technique enabled the conversion of a single-beam spectrometer into a pseudo-double-beam spectrometer [17]. Sample (S) and reference (R) cuvettes were placed on the same MIRE (covered or not with PEM). A computer-controlled lift serves for bringing the two chambers of the flow-through cell alternately into the path of the IR-beam for measurement (see supporting information, Section S1). Single channel spectra of reference and sample (from 4000 cm^−1^ to 800 cm^−1^ with 4 cm^−1^ spectral resolution) were measured separately one after another for parallel (p) and vertical (s) polarized light in the order: R (p), S (p), R (s), S (s) (measurement time ~142 s per set).

*PEM assembly on the MIRE (ATR crystal).* Prior to deposition of the PEM, the ATR crystal was cleaned with ultrapure water (Integra UV plus 2008-1, SG, elect. conductivity ≤ 0.6 μS/cm), ethanol (Sigma; grade reag. ISO, reag. Ph. Eur. ≥99.8% (GC)) and then chloroform (Fisher; HPLC grade stabilized with Ethanol (JT Baker)). For mechanical cleaning, soft laboratory wipes (Kimtech Science precision wipes) were used. Afterwards, the crystal was treated for 8 min in a plasma cleaner (PDC-326-2, 100 W, Harrick Plasma, Ithaca, NY, USA) to remove the remaining impurities and to render the surface more hydrophilic due to formation of hydroxyl groups and GeO_2_ [18]. The air pressure within the plasma cleaner was less than 0.07 mbar and the power transmitted to the radio-frequency coil was 18 W. The PEM coating was carried out by alternately dipping the ATR crystal into 0.5 mg/mL HA (Sigma-Aldrich, Taufkirchen, Germany) and 0.5 mg/mL PLL (Sigma-Aldrich, Taufkirchen, Germany) solutions in Tris buffer for 10 min with intermediate washing steps with the Tris buffer. A Tris buffer containing 10 mM Tris (Sigma, Rödermark, Germany) and 15 mM NaCl (Sigma, Rödermark, Germany) at pH adjusted to 7.2–7.4 using a hydrochloric acid (Merck, Darmstadt, Germany) was used. Polyethylenimine (PEI (Sigma-Aldrich, Taufkirchen, Germany), 0.5 mg/mL in Tris buffer) was used as an activating layer, providing a positive charge to the surface of the ATR crystal. Film build-up was conducted at 37 °C (in a water bath) for 8 cycles. Thus, 8 “bilayers” films were formed. Further, the following designation for the films (PLL/HA)_*i*_ will be used, where *i* defines the number of “bilayers” deposited during assembly of the PEM. Thus, for simplicity, it will not be specified that activating PEI was deposited at first.

*Procedure of the adsorption and desorption studies.* The Tris buffer solution was continuously passing through both (the reference (R) and sample (S)) chambers of a flow-through cell (see Section S1). Further, for adsorption studies, Lys (Calbiochem, Ontario, Canada) solution in the Tris buffer was passed through the sample chamber instead. Afterwards, for the desorption studies, the plain buffer flow was used again. The Lys supply lasted for ~20 h in the case of the PEM-coated crystal and ~14 h in the case of the blank crystal. Recording of spectra was performed for the first ~5 h in the case of the PEM-coated crystal and ~6.5 h in the case of blank crystal. The recording of spectra was renewed shortly prior to the change of Lys solution to the buffer for desorption studies. The duration of the measurements was chosen to be long enough to detect possible changes in the absorption spectra.

*Evaluation of the infrared spectra.* Prior to Fourier transformation, the Blackman–Harris three-term apodization with a zero-filling factor of 4 was performed. The mode “Power Spectrum” was used for phase correction. For this (initial) spectra treatment, the Software OPUS 6.0 (Bruker, Deutschland) was used. Further treatment was performed with the MATLAB software package. Protein absorption (AB) was calculated using the following equation:

AB = −lg (S/R).(1)

To exclude the influence of parameters which were considered to be unchanged with time (slight initial differences between the sample and the reference chambers may be due to, for instance, a difference in the rubber rings and/or PEM coverage), the spectrum taken prior to the protein supply to a blank or PEM-coated crystal was subtracted.

*Subtraction of water spectrum.* Negative absorbance was observed in the part of the amide I band from the side of higher wavenumbers for all studied cases: blank and PEM-coated crystals for both polarizations. Such spectra character is usually attributed to the fact that the peak of water banding vibration overlaps with the amide I band of proteins [19]. To take this into account, the protein/water absorption spectrum was recalculated based on the addition of a buffer/air absorption spectrum to the protein spectrum. The latter was assessed in such a way that there is a flat baseline in the range from 1720 cm^−1^ to 1850 cm^−1^ (for details, see Section S2) [19,20,21].

*Kinetics of Lys adsorption and desorption.* To follow the kinetics of adsorption and desorption of Lys on the blank and the PEM-coated crystal, the total absorbance of each absorption spectrum was calculated in the wavenumber range from 1480 cm^−1^ to 1720 cm^−1^ and normalized by the total absorbance of the last absorption spectrum obtained (see above *Procedure of the adsorption and desorption studies*).

*Spectrum of Lys in solution.* To obtain the spectrum of Lys in solution, the mean spectrum of the first seven spectra obtained after Lys solution replacement by buffer on the blank crystal was subtracted from the mean spectrum of the last seven spectra during the adsorption studies of Lys (for details, see Section 3.2 and Section S3). The mean values of several spectra were taken to decrease the noise.

*Spectrum of Lys released from the PEM.* To obtain a spectrum of the “poorly bound” fraction of Lys in the PEM (see Section S4), firstly, we calculated a mean spectrum of several spectra of Lys obtained during desorption when the quick release was terminated (see Section 3.2). Then, the resulting spectrum was subtracted from a mean spectrum of several Lys spectra obtained directly before the protein solution was replaced by plain buffer. The mean values of several spectra in both cases were taken to decrease the noise.

*Spectrum of Lys bound to the PEM.* To obtain a spectrum of the “tightly bound” fraction of Lys in the PEM, a mean spectrum of several spectra of Lys obtained during desorption when the quick release was terminated (see Section 3.2) was calculated. The mean value of several spectra was taken to decrease the noise.

*Area-normalization of the spectra.* For the sake of comparison, as well as calculation of the area overlap (see below *Calculation of area overlap*), the spectra were area normalized, i.e., rescaled, so that the area between the spectra and the baseline between 1353 and 2100 cm^−1^ was 10^4^. The range between 1353 and 2100 cm^−1^ was chosen to include the amide I and II bands. Additionally, the limits of the range chosen are sufficiently far away from, firstly, the amide I and II bands (not to be influenced by possible changes in the amide I and II bands of Lys) and, secondly, from the absorbance bands corresponding to water vapor (in order to exclude the possibility that the noise in the spectra arising from water vapor influences the result of area normalization of the spectra).

*Calculation of area overlap.* Two area-normalized spectra under comparison are plotted together in one graph. The 2D spaces under these two spectra for convenience of description are referred to as Sp1 and Sp2. To calculate the area overlap (in %) between Sp1 and Sp2, the following equation was applied:

(2) $$AreaOverlap=\frac{2\cdot A-AreaLack}{2\cdot A}\cdot 100\%,$$

where *A* is the area under the area-normalized spectra in the chosen wavenumber range (here, *A* = 10^4^, as mentioned above). *AreaLack* is the area of the parts under the spectra, where Sp1 and Sp2 do not overlap, which can be approximated as follows:

(3) $$AreaLack=\overset{WN_{end}}{\underset{i=WN_{start}}{\sum }}\left|Abs_{i}^{1}-Abs_{i}^{2}\right|\cdot h,$$

where $WN_{start}$ and $WN_{end}$ are the limits of the wavenumber region where the spectra have an equal area (here 1353 and 2100 cm^−1^, respectively); $Abs_{i}^{1}$ and $Abs_{i}^{2}$ are the absorbances of the two spectra at wavenumber *i*; *h* is the wavenumber interval (here, 0.96 cm^−1^).

## 3. Results and Discussion

### 3.1. Study of Lys Adsorption on the Blank and PEM-Coated Crystal

The adsorption of Lys on a PEM-coated Ge-crystal was studied via polarized ATR-FTIR spectroscopy. To achieve this, the ATR crystal was covered with a (PLL/HA)_8_ film. Lys solution (1 mg/mL) in Tris buffer was continuously supplied into a flow-through cell. Measurements for p and s polarization were performed. Additionally, for comparison, separate measurements were performed to investigate the adsorption of Lys on a blank Ge-crystal.

**Figure 1:**
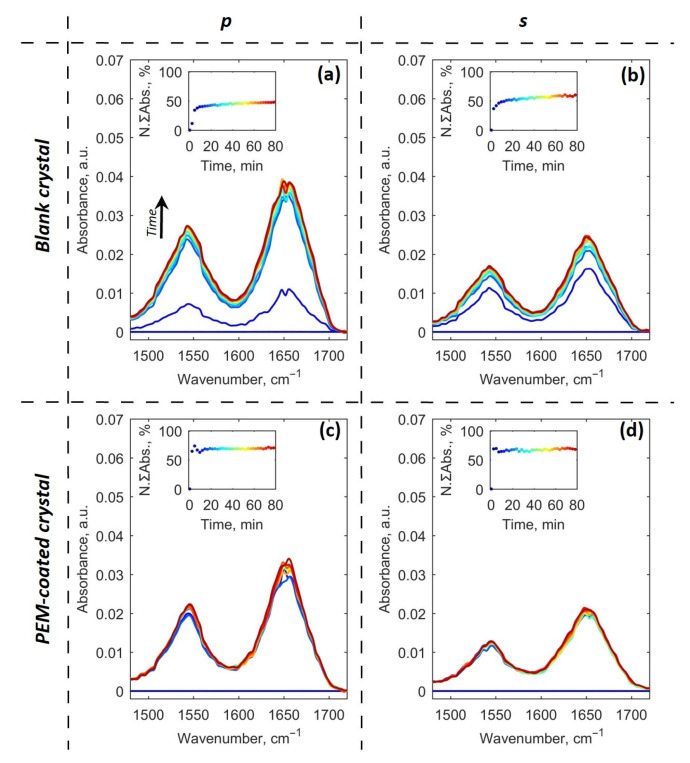
ATR-FTIR spectra recorded upon adsorption of Lys on the blank (**a**,**b**) and the PEM-coated (**c**,**d**) crystal for p (**a**,**c**) and s (**b**,**d**) polarization. The black arrow in *a* indicates the direction of the time span. Inset graphs represent the temporal evolution of the total absorbance normalized by the total absorbance of the last spectrum recorded during adsorption studies (see Materials and Methods (Section 2)). The colors of the presented data (from cold to warm) reflect the direction of the time span. The colors of the spectra correspond the colors of the data in the inset graphs.

Figure 1 shows sequential absorption spectra of Lys between 1480 and 1720 cm^−1^ during 80 min after addition of Lys to the PEM. The inset graphs in Figure 1 represent the kinetics of the temporal development of the total absorbance in the range between 1480 and 1720 cm^−1^.

The characteristic absorbance bands (amide I and amide II) in Figure 1 show an increasing amount of the protein within the sampling depth of the evanescent wave [22]. As can be seen from the inset graphs, in both cases, for the PEM-coated and the blank crystal, a rapid increase in absorbance during the first stage of protein adsorption turns to a much slower, gradual increase for longer time periods. The specific changes in the spectra will be discussed in more detail in Section 3.3 and Section 3.4.

**Scheme 1:**
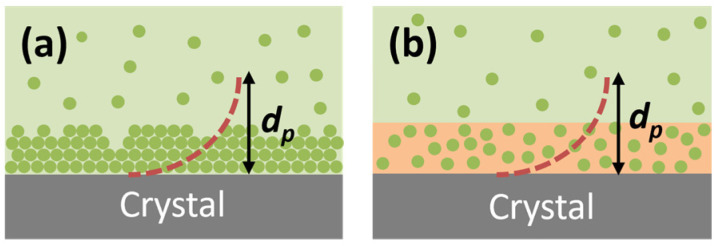
Schematics of the protein (the green spheres) adsorption on the blank (**a**) and PEM-coated (**b**) crystal. The dashed line represents the evanescent wave characterized by a penetration depth *d_p_*. The scheme illustrates the different character of Lys adsorption on the blank and PEM-coated crystal and is not to scale. In reality, Lys does not have a spherical but rather an ellipsoidal shape, and it may form less homogeneous layers than presented in (**a**) [25,27].

The thickness of the sample on the ATR crystal (in the present study of the protein layer or the PEM film), in particular, its relation to the penetration depth of the evanescent wave ($d_{p}$), plays an important role in the interpretation of the data. It is easier to start with the case of the blank crystal. It is known that Lys adsorption produces multilayers (Scheme 1a) [23] if the protein concentration is sufficiently high, as it is the case in this experiment. For instance, formation of around 16 layers of Lys on Ge crystal was reported upon 8 h of adsorption performed at conditions similar to those in the present study [24]. This corresponds to a total thickness of around 50–70 nm (molecular dimensions of Lys are 45 × 30 × 30 Å [23,25]). This value is far below the penetration depth of the evanescent wave. For our conditions: with a Ge crystal and angle of incidence of 45°, the penetration depth is around 428 nm in the amide II region (at 1550 cm^−1^), as shown in the following equation [26]:

(4) $$d_{p}=\frac{\lambda }{2\pi n_{1}\sqrt{sin^{2}\theta -{\left(n_{2}/n_{1}\right)}^{2}}},$$

where λ is the wavelength; $n_{1}$ and $n_{2}$ are the refractive indices of the crystal and of the medium within the field distribution of the evanescent wave, respectively; θ is the angle of incidence.

The thickness of the PLL/HA PEMs of 8–10 bilayers is reported to be around 100–200 nm [28]. Thus, the thickness of the PEM on the crystal is also below the penetration depth of the electromagnetic evanescent field. It is noteworthy that, as shown in our previous reports [29,30], protein molecules penetrate the interior of the films to achieve homogenous distribution therein (Scheme 1b). Additionally, the thickness of the PEM does not change upon protein adsorption [30].

It is important to note that for both blank and PEM-coated crystal, the signal in case of p polarization is noticeably stronger than that in case of s polarization (compare the main graphs in Figure 1a–d). According to theory, for isotropically oriented samples, the signal of p polarization is always stronger than that of s polarization. This phenomenon can be explained based on a concept of the so-called effective thickness introduced by Harrick [31]. The effective thickness is the thickness of a layer which has the same absorbance in transmission spectra at normal incidence as that found in ATR [32]. The effective thickness for s ($d_{es}$) and p ($d_{ep}$) polarizations can be written as:

(5) $$d_{es}=\frac{2n_{21}d_{p}cos\theta }{1-n_{21}^{2}},$$

(6) $$d_{ep}=\frac{2n_{21}d_{p}cos\theta \left(2sin^{2}\theta -n_{21}^{2}\right)}{\left(1-n_{21}^{2}\right)\left(\left(1+n_{21}^{2}\right)sin^{2}\theta -n_{21}^{2}\right)},$$

where $n_{21}$ is $n_{2}/n_{1}$.

Based on these equations, it follows that at an angle of incidence of 45°, the effective thickness for p polarization is nearly twice that for s polarization. In Figure 1, the single exception is the first spectrum recorded during protein adsorption onto the blank crystal (compare the main graphs in Figure 1a,b) where the absorbance for s polarization is stronger than that for p polarization. This fact may be attributed to the delay in time (of around 1 min 11 s, see Materials and Methods (Section 2)) between recording the spectra of s and p polarization. During the first stage of protein adsorption, such a delay leads to a considerable difference in the amount of protein adsorbed.

### 3.2. Study of Lys Retention on the Blank and PEM-Coated Crystal

**Figure 2:**
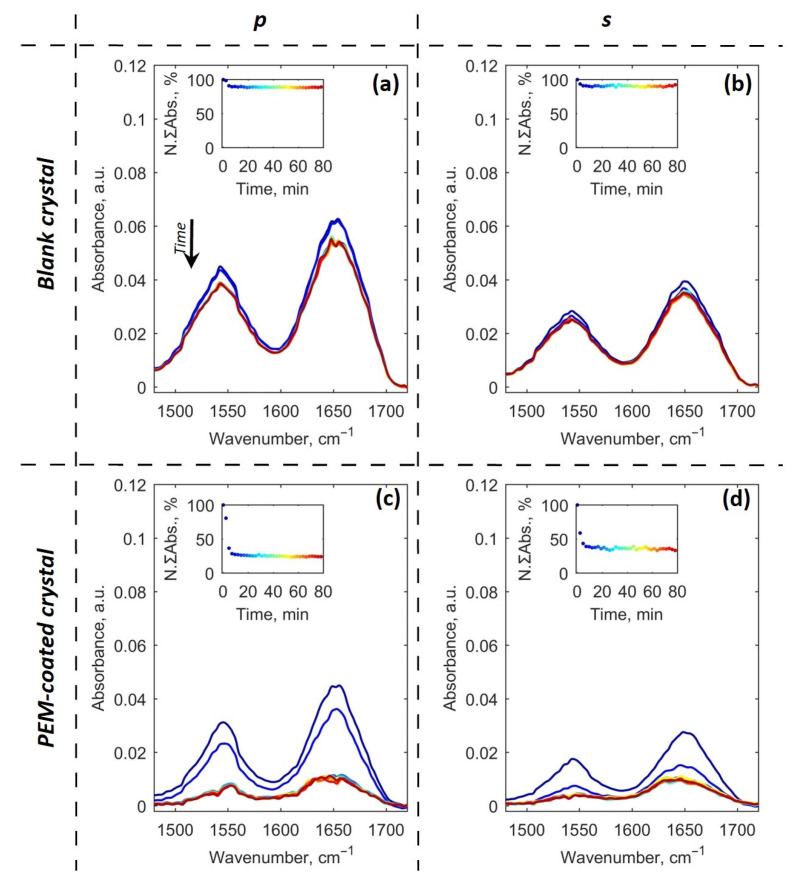
ATR-FTIR spectra recorded during desorption studies of the protein when the protein solution was replaced by buffer. Data are shown for the blank (**a**,**b**) and PEM-coated (**c**,**d**) crystal for p and s polarizations (respectively). The arrow in (**a**) indicates the direction of the time span. Inset graphs show the time course of the total absorbance normalized by the total absorbance of the last spectrum recorded during adsorption studies. The colors of the data (from cold to warm) reflect the direction of the time span. The colors of the spectra are in accordance with the colors of the data in the inset graphs.

After protein adsorption, the protein solution was replaced by a buffer solution to assess the retention of the protein in the PEM-coated crystal and on the blank crystal. Figure 2 shows the evolution of spectra upon buffer supply. Inset graphs represent the kinetics of change of the total absorbance of spectra. The latter is normalized by the total absorbance of the last spectrum recorded prior to the buffer supply (see Materials and Methods (Section 2)).

A minor decrease in absorbance is observed for the blank crystal (Figure 2a,b). In contrast, for the PEM-coated crystal, the decrease in the signal is considerable (Figure 2c,d). For both the blank and the PEM-coated crystal, the decrease in absorbance with time demonstrates a decrease in the amount of the protein found within the sampling depth of the evanescent wave. The decrease may have two causes. It can be attributed to the desorption of the protein from the surface of the blank crystal or from the PEM (“desorption factor”) and to the replacement of the protein solution by buffer (“solution factor”).

Formation of strongly bound, sticky layers of Lys on crystals is a well-known phenomenon [20,25]. Literature reports state that the longer the adsorption time is, the higher the amount of the irreversibly bound Lys is [23]. Notably, in contrast to the adsorbed Lys layers in the immediate vicinity to the crystal, layers further away and closer to the solution phase do not experience considerable conformational changes [24]. This allows one to obtain the spectrum of Lys in solution through subtraction of the spectrum of the adsorbed protein layer in the buffer (start of desorption studies) from the spectrum of the adsorbed protein layer in the protein solution (end of adsorption studies). A difference spectrum obtained in such way will be used as a reference spectrum for further analysis of the data (see Section S3).

In case of the PEM-coated crystal, the contribution of “the solution factor” in the overall measured signal cannot be higher than that in the case of the blank crystal, as the thickness of the PEM is larger than the thickness of the protein layer adsorbed on the blank crystal. In other words, “the solution factor” alone cannot explain the considerable drop in the absorbance in the case of the PEM (Figure 2c,d). Thus, in the case of the PEM-coated crystal, the “desorption factor” is primarily responsible for the observed change in the signal. This means that considerable desorption of the protein from the PEM takes place. Consequently, one can distinguish between two Lys fractions in the PEM: A “poorly bound” and a “tightly bound” one. The “poorly bound” fraction represents around 60–70% of the overall amount of the protein adsorbed by the PEM and is released from the PEM upon washing with buffer (see insets in Figure 2c,d). The other 30–40% of Lys correspond to the “tightly bound” fraction, which remains in the PEM for the time periods investigated.

### 3.3. Comparison of Lys Spectra on the Blank Crystal and in Solution

To compare spectra of Lys adsorbed into the blank crystal (and further the spectra of Lys adsorbed into the PEM-coated crystal, see Section 3.4) with the spectrum of Lys in solution we follow the strategy suggested by van de Weert et al., 2001 [14]. Firstly, two spectra under comparison are normalized, such that areas under each spectrum within a given spectral range equal each other. Secondly, the resulting area-normalized spectra are plotted together and the percentage of overlapping area between them (further called *area overlap*) is calculated (see Section 2). Notably, area overlap values of around 95% point to a high degree of resemblance of the spectra under comparison. Area overlap values below 85% point to a poor correlation between the spectra [14].

#### Lys on the Blank Crystal

**Figure 3:**
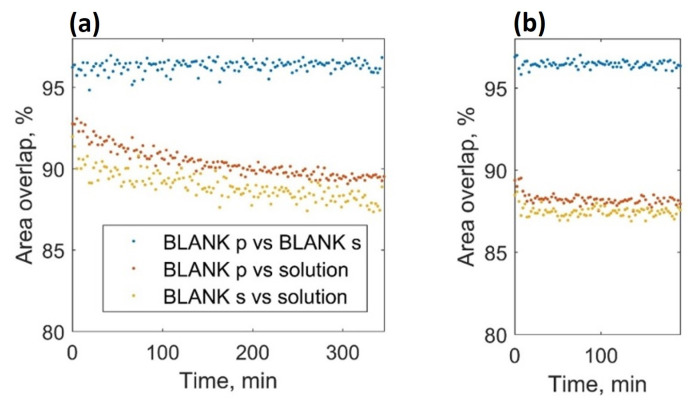
Time course of area overlap between the spectrum of Lys adsorbed to the blank crystal and spectrum of Lys in solution during adsorption (**a**) and desorption (**b**).

Area overlap between the spectrum of Lys on the blank crystal and the spectrum of Lys in solution for p and s polarization (Figure 3, data in red and yellow, respectively) was calculated for various time periods elapsed during Lys adsorption and desorption. In addition, the area overlap between the spectra of Lys recorded with p and s polarization on the blank crystal was calculated (Figure 3, data in blue).

At the beginning of the adsorption studies, values for the area overlap between Lys spectra on the blank crystal and in solution were around 92.7 ± 0.3 and 90.8 ± 0.9% for p and s polarizations, respectively. The decrease in the area overlap values upon Lys adsorption suggests that the structure of Lys adsorbed to the crystal changes and its correlation to the structure of Lys in solution degrades. At the beginning of the desorption studies area overlap values slightly decrease down to 88.4 ± 0.2 and 87.5± 0.3% for p and s polarizations, respectively.

The area overlap values observed for p and s polarizations are very similar (data in red and yellow, Figure 3). This suggests that there is no preferential orientation of the secondary structure elements of Lys on the crystal surface. Indeed, the area overlap values calculated for spectra of p polarization versus those of s polarization (data in blue, Figure 3) lay at around 96.1 ± 0.2% at the beginning of the adsorption studies and remain unchanged. The lack of a preferential orientation of Lys on the crystal is in line with previous literature reports [3,33].

**Figure 4:**
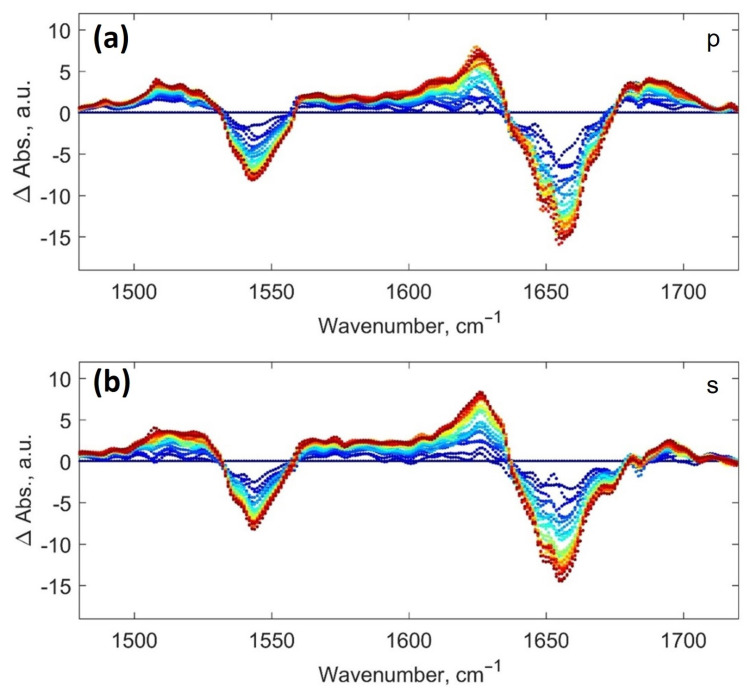
Difference between spectra recorded during Lys adsorption on the blank crystal compared to the first spectrum of Lys on the blank crystal for p (**a**) and s (**b**) polarizations. The change in color of the data presented from cold to warm reflects the direction of the time span. Mean values in each case of five spectra are taken to decrease the noise. The time interval between the presented spectra is around 13 min.

It is of interest to have a closer look at the spectral changes taking place over time. To analyze the changes, an approach called difference spectroscopy was applied. The spectrum recorded at the beginning of the adsorption studies was subtracted from the subsequent spectra. The resulting spectra (difference spectra) are represented in Figure 4a,b for p and s polarizations, respectively.

Based on Figure 4, one may see that the spectra experience considerable gradual changes over time. A decrease in absorbance takes place at around 1543 and 1653 cm^−1^, and an increase in absorbance takes place at around 1626 cm^−1^. A less considerable increase in absorbance occurs in the wavenumber regions between 1500 and 1525 cm^−1^, between 1560 and 1610 cm^−1^, as well as at around 1695 cm^−1^.

There are two factors which can contribute to the observed changes in the spectra. Firstly, upon Lys adsorption, dehydration of Lys (factor 1) may take place [14]. This process can be accompanied by a broadening of amide I and II bands in the protein spectra. In the difference spectra, such changes would result in a decrease in absorbance at the maxima of amide I and II bands, as well as an increase in absorbance in both wings of the amide bands (Figure 4). Secondly, upon Lys adsorption, modifications of its secondary structure may take place (factor 2). It is known that the amide I band is more sensitive to the secondary structure than the amide II band [14]. The fact that modifications of the amide I differ from those in the amide II band (Figure 4) suggests that the secondary structure of Lys changes. Specifically, the increasing bands at around 1626 cm^−1^ and 1695 cm^−1^ may indicate formation of antiparallel beta sheets or aggregated strands. The latter changes may take place at the expense of a decrease in the amount of helical motifs. Indeed, the decreasing absorbance at 1543 and 1653 cm^−1^ can be attributed to a loss of helical structures. Notably, the observed changes are in line with previous reports summarized, for instance, in Ref. [14].

### 3.4. Comparison of Lys Spectra on the PEM-Coated Crystal and in Solution

#### 3.4.1. Analysis Based on Area Overlap Values

**Figure 5:**
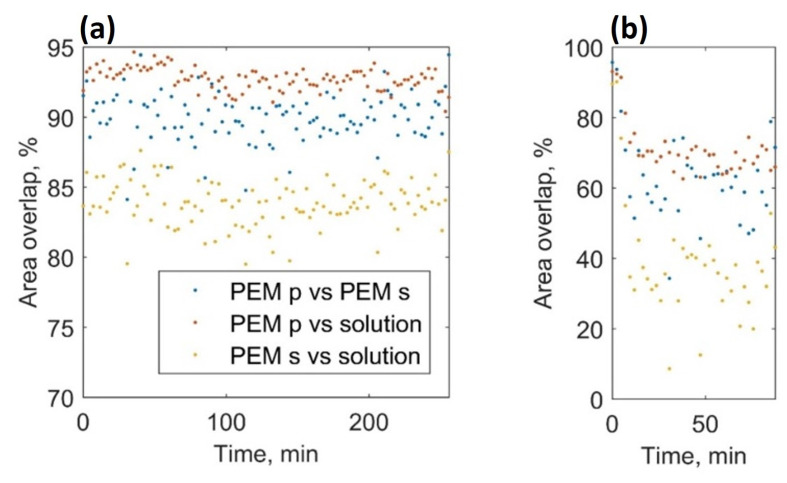
Time course of area overlap between spectrum of Lys loaded to the PEM-coated crystal and spectrum of Lys in solution during adsorption (**a**) and desorption (**b**).

The area overlap between the spectrum of Lys on the PEM and the spectrum of Lys in solution was calculated and is presented for various time periods during Lys adsorption and desorption into and from the PEM for p and s polarization (Figure 5, data in red and yellow, respectively). In addition, the area overlap between the spectra of Lys in the PEM recorded with p and s polarizations was calculated (Figure 5, data in blue). One may note a slight nonmonotonic behavior of the area overlap values during adsorption (Figure 5a). This can be seen more clearly in the data obtained with p polarization versus solution (Figure 5a, data in red). Specifically, a decrease in area overlap values takes place after around 70 min after the start of the adsorption studies. This observation will be discussed in more detail in the next section.

At the beginning of the desorption studies, area overlap values drop down from 91.9 ± 1.1 to 69.3 ± 1.1 and from 84.5 ± 1.8% to 34.7 ± 6.0% for p and s polarizations, respectively (Figure 5b, data in red and yellow). Such a considerable decrease in the area overlap values during desorption suggests that the spectra of Lys released from the PEM and Lys loaded into the PEM considerably differ. For the “tightly bound” fraction of Lys, a poor correlation with the structure of Lys in solution can be concluded based on the area overlap values specified above (below 70% for both polarizations, see Figure 5b). Additionally, the area overlap between the spectra corresponding to the “tightly bound” fraction recorded with p and s polarizations is only around 60% (Figure 5b, data in blue). This points to a certain degree of orientation of the “tightly bound” Lys in the PEM. The issue of orientation of Lys in the PEM will be discussed in more detail below (see Section 3.4.3).

One key experiment of this work is the characterization of the “poorly bound” fraction of Lys in the PEM. This refers to the fraction of Lys which is quickly released from the PEM after replacing the protein solution by plain buffer. To obtain a spectrum of the “poorly bound” fraction of Lys in the PEM (see Section S4), firstly, a mean spectrum of several spectra of Lys obtained when the quick release was terminated (see inset graphs in Figure 2c,d) was calculated. Then the resulting spectrum was subtracted from a mean spectrum of several Lys spectra obtained directly before the protein solution was replaced by plain buffer. The mean values of several spectra in both cases were taken to decrease the noise. The area overlap values for the area-normalized spectrum of the released Lys with the spectrum of Lys in solution were calculated and were found to be 95.5 and 93.5% for p and s polarizations, respectively. Thus, the structure of Lys released from the PEM is close to the structure of Lys in solution. The close area overlap values obtained for the two polarizations indicate that the “poorly bound” Lys is randomly oriented within the PEM. The area overlap between the spectra of the released Lys recorded with p and s polarization was calculated and was found to be 98.7%. This indicates a high degree of resemblance between these spectra and points to a random orientation of the “poorly bound” fraction of the protein in the PEM.

#### 3.4.2. A Peculiarity Observed upon Lys Adsorption into the PEM

**Figure 6:**
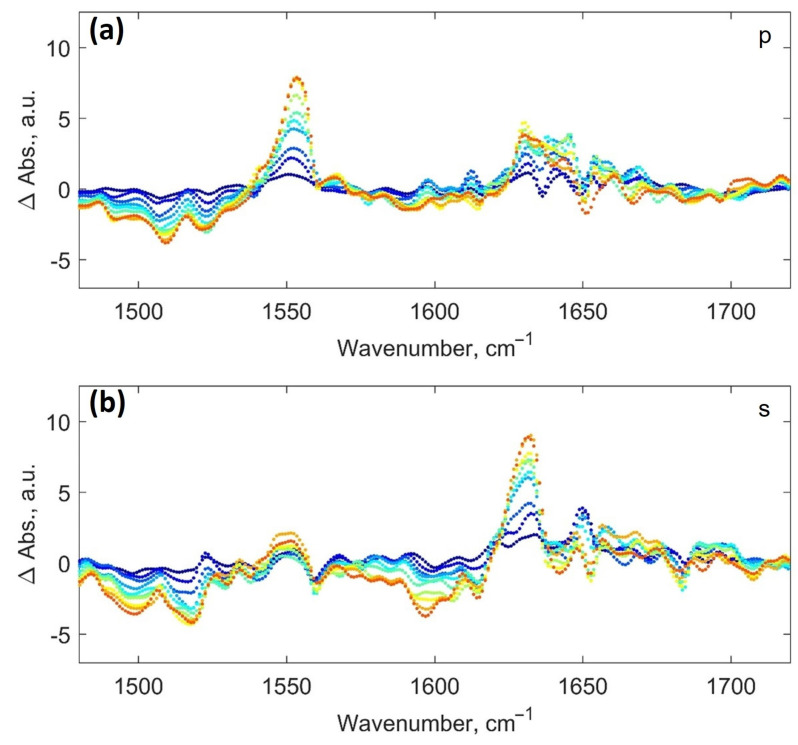
Difference between spectra recorded during Lys adsorption into the PEM during the time interval between 70 and 90 min and the spectrum of Lys taken during adsorption of Lys into the PEM after around 65 min for p (**a**) and s (**b**) polarizations. The changes in color of the presented data from cold to warm reflect the direction of the time span. Each of the spectra is obtained from sampling five spectra to decrease the noise. Time intervals between the presented spectra are around 2.37 min.

It is of interest to analyze which changes take place in the Lys spectra during adsorption into the PEM in the time region 70–90 min, which induced the decrease in the area overlap values presented in Figure 5. To achieve this, the approach of difference spectroscopy, similar to the case of the blank crystal (see Section 3.3, Figure 4), was applied. The spectrum recorded at 65 min after start of the adsorption studies was subtracted from the spectra recorded in the time region 70–90 min. The resulting spectra (difference spectra) are represented in Figure 6 for p and s polarizations, respectively.

Firstly, it is worth noting that the difference spectra presented in Figure 6 are noisier compared to the difference spectra in case of the blank crystal (Figure 4). Still, in Figure 6, a remarkable increase in absorbance can be seen at around 1550 cm^−1^ for p polarization and at around 1631 cm^−1^ for the s polarization. Since the changes observed are different for p and s polarizations, some reorganization—including reorientation of Lys—may take place in the PEM during this time span. Changes observed at 1630 and 1550 cm^−1^ are most likely a result of the formation/increase in the amount of parallel beta sheet structures [34,35] with the beta strand axis oriented perpendicular to the surface of the PEM [36,37] (for details, see Section S5).

Apart from spectral changes at 1550 and 1631 cm^−1^, in Figure 6, one may note a decrease in absorbance taking place below 1540 cm^−1^, as well as between 1560 and 1620 cm^−1^ for both polarizations. The former changes can be interpreted as a decrease in the number of helix motifs that show a band at around 1520 cm^−1^. Changes in the right wing of the amide II band can be related to changes in presentation of the side chains of the protein (aspartate and glutamate side chains absorb at around 1584 cm^−1^). Additionally, for s polarization, a decrease in absorbance can be noted around 1645–1655 cm^−1^. These changes can be related to a decrease in alpha helical structures. For p polarization changes also take place between 1635 and 1675 cm^−1^. However, there is no distinct trend in this region.

#### 3.4.3. Conformation and Orientation of Lys in the PEM

Results presented above suggest the presence of two Lys fractions in the PEM: A “poorly bound” (subsequently released from the PEM) and “tightly bound” (remaining in the PEM) fraction. Area overlap values (see Section 3.4.1) indicate that, in contrast to the released Lys, Lys remaining in the PEM appears to possess a specific orientation in the PEM and its conformation considerably deviates from that of native Lys in solution. It may be of interest to have a closer look at the spectra of Lys in the PEM, especially of the adsorbed Lys fraction. The issue of resemblance of Lys spectra in the PEM to the Lys spectrum in solution, as well as the issue of Lys orientation in the PEM, will be addressed.

**Figure 7:**
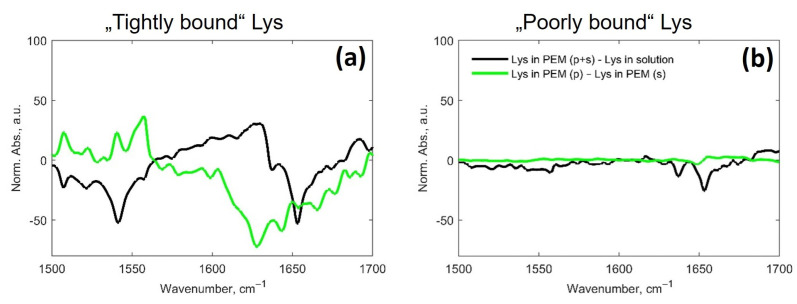
The spectra in black represent the difference between the area-normalized spectrum of the “tightly bound” fraction of Lys in the PEM or “poorly bound” fraction and the spectrum of Lys in solution (*a* or *b*, respectively). Spectra in green represent the difference between area-normalized p and s polarizations spectra of the “tightly bound” (**a**) and the “poorly bound” (**b**) fractions of Lys.

To analyze the orientation of Lys in the PEM, the area-normalized spectrum of Lys remaining in the PEM recorded with s polarization was subtracted from that recorded with p polarization (see the difference spectrum in green in Figure 7a—also called a *dichroic spectrum*). It is worth noting that a very similar dichroic spectrum can be obtained from the initial (not area-normalized) spectra of Lys (for details, see Section S6). In addition, our study analyzed how the structure of Lys remaining in the PEM differs from that of Lys in solution. In the first step, a spectrum of Lys remaining in the PEM including both polarizations was obtained. To achieve this, the initial (i.e., not area-normalized) spectrum of Lys remaining in the PEM recorded with parallel (p) polarization was added to that recorded with perpendicular (s) polarization multiplied by a certain factor R^iso^ [38]. This is called a dichroic ratio for an isotropic sample, and for our experimental conditions it is equal to 1.93 [39]. In a second step, the spectrum obtained was area-normalized. Finally, a difference between the resulting spectrum and the area-normalized spectrum of Lys in solution was calculated (see the difference spectrum in black in Figure 7a). Additionally, in Figure 7b, corresponding difference spectra are presented for Lys released from the PEM (which does not show a specific orientation, see Section 3.4.1) for comparison.

The spectrum of Lys remaining in the PEM shows considerable deviation from the spectrum of Lys in solution compared to the case of the spectrum of released Lys (compare the spectra in black in Figure 7a,b). A much higher difference between the spectra recorded with p and s polarizations can be also observed for the case of Lys remaining in the PEM compared to the case of the released Lys (compare the spectra in green in Figure 7a,b). Thus, the difference spectra presented in Figure 7 support the result obtained by the analysis of the area overlap values (see Section 3.4.1).

It is of interest to analyze the spectra corresponding to the case of Lys remaining in the PEM in more detail (Figure 7a). In Figure 7a, one may note a strong correlation in the position of the peaks between the black and green difference spectra. In the black difference spectrum, the largest peaks pointing downward at 1541 and 1653 cm^−1^, as well as that at 1522 cm^−1^, may indicate a lower number of alpha helical structures in Lys remaining in the PEM compared to Lys in solution [11,40,41,42]. In the difference spectrum in green, the bands at 1522 cm^−1^ and 1541 cm^−1^ are pointing upward, whereas the band at 1653 cm^−1^ is pointing downward. These observations may indicate an orientation of the helix axis close to normal to the plane of the crystal (and the PEM), as in an alpha helix the amide I dipole moment is close to the helix long axis whereas the amide II dipole moment is roughly perpendicular to it [15].

In the black difference spectrum, the strongest positive peak observed at 1631 cm^−1^ indicates a higher number of beta sheet structures in Lys remaining in the PEM compared to Lys in solution. Absorbance at around 1630 cm^−1^ may be characteristic for both parallel and antiparallel beta sheets (as mentioned above). The presence of the parallel beta sheets in the black difference spectrum is reflected in a slight positive peak at around 1552 cm^−1^ (arising after around 70 min from the start of the adsorption). Notably, a peak at this wavenumber (1552 cm^−1^) can be also seen in the difference spectrum in green as a shoulder of a stronger positive peak observed at 1557 cm^−1^. Additionally, in the black difference spectrum, a peak at 1695 cm^−1^ characteristic for antiparallel beta sheets [35] can be seen. These beta sheets, in contrast to the parallel beta sheets, are apparently present in Lys adsorbed to the PEM throughout the observed desorption period. Thus, the difference spectra presented indicate the presence of a higher number of both parallel and antiparallel beta sheets in Lys remaining in the PEM compared to Lys in solution. Finally, a peak observed at 1557 cm^−1^ (which is especially strong in the difference spectrum in green), as well as that at 1573 cm^−1^ and those between 1665 and 1695 cm^−1^ are assigned to turn structures [42].

In summation, considerable differences in the number of alpha helices, beta sheets and turns structures in Lys remaining in the PEM compared to Lys in solution can be concluded. The dichroic spectrum presented (the difference spectrum in green in Figure 7a) indicates that these secondary structure elements have a specific orientation in Lys remaining in the PEM. The presence of a specific orientation of Lys in the PEM can be related to macromolecular interactions within the PEM. The layered character of adsorption of the polyelectrolyte “layers” during the assembly of the PEMs can play a role. However, it should be stressed that it is generally accepted that this type of PEM is characterized by a strong interpenetration of the PEs and does not possess a strict layered structure [43]. In addition, in the immediate vicinity of the crystal surface, the polymer chains may be less flexible and, together with the hard surface of the crystal, may lead to nonreversible adsorption.

### 3.5. Lys on the PEM-Coated Versus Blank Crystal

**Figure 8:**
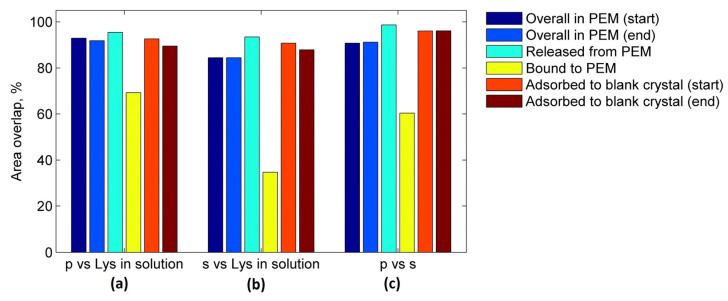
The bars in (**a**,**b**) represent area overlap values between spectra of Lys in case of the PEM-coated or blank crystal and the spectrum of Lys in solution for p and s polarizations, respectively. The bars in (**c**) represent area overlap values between spectra of Lys in case of the PEM-coated or blank crystal recorded with p and s polarizations. The cases “Overall in PEM (start) and (end)” as well as “Adsorbed to blank crystal (start) and (end)” refer to the stage of Lys adsorption (absorption spectra recorded at the beginning and at the end of the adsorption studies, respectively).

It is informative to perform a comparison of the results obtained for the cases of the PEM-coated and blank crystals. In Figure 8, the area overlap values obtained for the cases of the PEM-coated and the blank crystal are shown for comparison. One may see that spectra of the protein bound to the PEM differ the most from the protein spectrum in solution. The largest difference between the spectra recorded with p and s polarizations is also observed for the Lys fraction remaining in the PEM. The protein released from the PEM shows the highest degree of resemblance to Lys in solution and the lowest degree of orientation compared to the other cases.

## 4. Conclusions

One of the central questions for the application of PEMs is whether the secondary structure of biomolecules loaded into the films is retained. In the present work, this issue is addressed by investigating the interaction of Lys with PLL/HA multilayers using polarized ATR-FTIR spectroscopy.

The present study suggests that Lys exhibits different properties in terms of adsorption/desorption kinetics, conformation and orientation on the two types of crystal surfaces used—Ge crystal coated with a PEM or uncoated. On both PEM-coated and blank crystal surfaces, Lys experiences a conformational change. In both cases, reduced alpha helical content and a higher number of beta sheets compared to the native Lys in solution is observed. In the case of the blank crystal, the spectra show gradual changes upon Lys adsorption indicating (apart from conformational changes) a dehydration of Lys. Presented data evidence the formation of a strongly bound Lys layer on the blank crystal. A high degree of resemblance between the absorption spectra recorded with p and s polarized infrared light point to a lack of a specific orientation of Lys on the blank crystal. Results obtained for Lys on the blank crystal are in line with previous literature reports on Lys adsorption kinetics [24], conformation [14,20] and orientation [3,33].

In the case of the PEM-coated crystal Lys appears to modify its conformation immediately during adsorption. Additionally, approximately 70 min after the adsorption begins, the formation of parallel beta sheets is detected. Desorption studies indicate the presence of a “tightly bound” Lys fraction remaining in the PEM, and a “poorly bound” one, which is released from the film. The latter represents around 60% of the overall amount of the loaded Lys and reflects a high degree of resemblance to the spectrum of Lys in solution. The “tightly bound” fraction, in contrast, considerably differs from that for Lys in solution. Additionally, considerable differences between the spectra of the “tightly bound” Lys recorded with p and s polarized infrared light are observed. Analysis of the dichroic spectrum of the “tightly bound” Lys points to the presence of a specific orientation of alpha helical structures, beta sheets and turns.

The results obtained on Lys adsorption/desorption kinetics, conformation and orientation in the PEMs are important for practical applications of the PEMs. PEMs can serve as a platform for controlled release of protein-based bioactives such as growth factors, hormones, cytokines e.g., in the field of tissue engineering or cell culture. Finally, it is demonstrated that the application of ATR-FTIR technique represents a powerful approach that can be used not only to study the secondary structure of proteins loaded in PEMs, but also to investigate the kinetics of protein adsorption and desorption in order to reveal mechanisms relevant to loading and release of proteins. In future work, we would like to use alternative techniques to better understand the phenomena observed in this study and also ensure the reproducibility of the findings.

## Data Availability

Data supporting the findings of this study are available from the corresponding author upon reasonable request.

## Supplementary Materials

The following supporting information can be downloaded at: https://www.mdpi.com/article/10.3390/polym15041036/s1, Figure S1: Components of the flow-through cell used for the ATR-FTIR measurements (adapted from [16]): (a) a photo of assembled flow-through cell; (b) a photo of two side panels of the flow-through cell (reference and sample chambers are separated through the viton rings; through the tubes solutions can be independently passed to the reference and sample chambers); (c) a scheme reflecting that through a lift the whole flow-through cell can be moved up and down (as a result either the sample or the reference chamber can reach the path of the IR beam); Figure S2: Example spectrum of Lys before and after water correction and spectrum of water. Presented Lys spectra correspond to the case of Lys on the blank crystal at the beginning of adsorption for p polarization; Figure S3: Comparison of the area-normalized difference spectrum with the area-normalized spectrum of the protein adsorbed to the blank crystal for the case of p (a) and s (b) polarization. (c) Comparison of the area-normalized difference spectrum in case of p and s polarization; Figure S4: The area-normalized difference spectrum compared to the area-normalized spectrum recorded just after start of Lys delivery to the blank crystal for the case of p (a) and s (b) polarization; Figure S5: The area-normalized spectrum of Lys released form the PEM compared to the are-normalized spectrum of Lys in solution; Figure S6: Dichroic spectrum of Lys adsorbed to the PEM obtained as p- minus s-spectrum after multiplication of the s-spectrum by 1.93. References [15,16,19,20,21,34,35,36,37,39,40,44,45,46] are cited in the supplementary materials.
