# Supplementary material for: Interaction of Lysozyme with Poly(L-lysine)/Hyaluronic Acid Multilayers: An ATR-FTIR Study
Source: Polymers (Basel). 2023 Feb 19;15(4):1036. doi: 10.3390/polym15041036 (PMC9964902; doi:10.3390/polym15041036)
Supplement: Supplementary file 1 [file polymers-15-01036-s001.zip › polymers-2163626-supplementary.pdf]

Supporting information

Section S1. Design of the flow-through cell used for the ATR-FTIR measurements

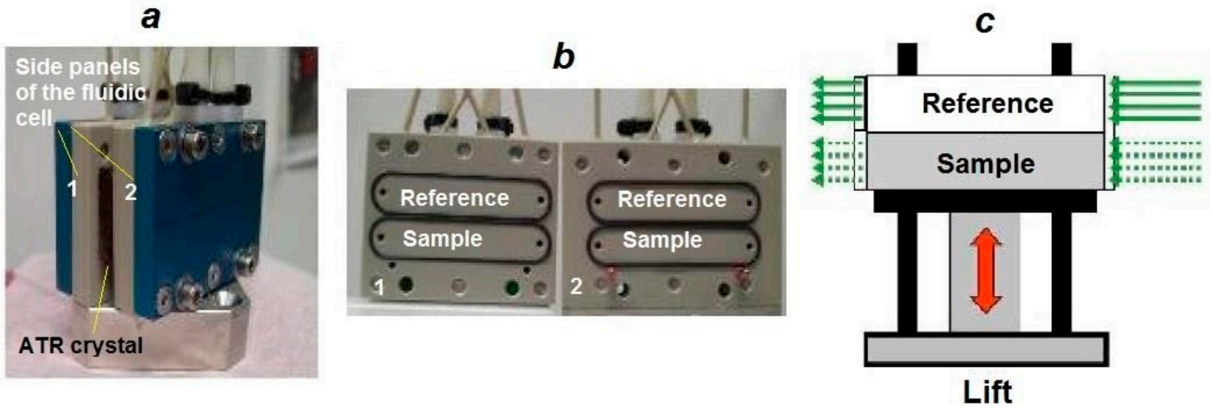

Figure S1. Components of the flow-through cell used for the ATR-FTIR measurements (adapted from [16]): (**a**) a photo of assembled flow-through cell; (**b**) a photo of two side panels of the flow-through cell (reference and samplechambers are separated through the viton rings; through the tubes solutions can be independently passed to the reference and sample chambers); (**c**) a scheme reflecting that through a lift the whole flow-through cell can be moved up and down (as a result either the sample or the reference chamber can reach the path of the IR beam).

Section S2. Subtraction of water spectrum

As mentioned in the experimental section, negative absorbance was observed in the part of the amide I band from the side of higher wavenumbers for all studied cases: blank and PEM-coated crystal for both polarizations (Figure S2). Such spectra character is a widely known phenomenon which is usually attributed [19] to the following effect. Protein absorption spectra are calculated using water (or buffer) [40] solutions as reference (see Eq. (1) in the main text). However, the refractive index of a protein solution is higher than that without the protein and increases upon increase of the protein concentration [20]. This leads to a lower penetration depth of the evanescent wave (see Eq. (4) in the main text) in case of solution with protein than in the case of pure buffer. As a result, water absorbance of solution with protein is slightly lower than that of solution without the protein. Since the banding vibration peak of water centered around 1645 cm^{-1} [44] overlaps with the amide I band of proteins and is much broader than the latter (Figure S2), the described effect is pronounced in the spectral range from 1720 to 1850 cm^{-1}. This allows one to account for this effect and recalculate the protein/water absorption spectrum by addition to the protein spectrum a buffer/air absorption spectrum. The latter should be weighted in such a way that a flat baseline is achieved in the range from 1720 to 1850 cm^{-1} [19, 20,21]. As mentioned in the experimental section, such correction has been performed (Figure S2).

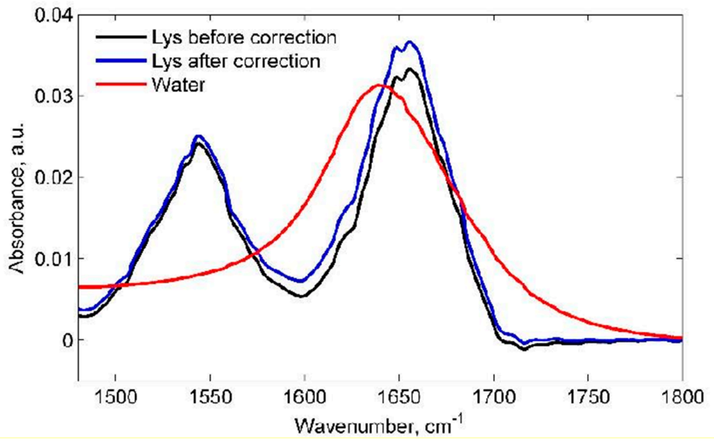

Figure S2. Example spectrum of Lys before and after water correction and spectrum of water. Presented Lys spectra correspond to the case of Lys on the blank crystal at the beginning of adsorption for p polarization.

Section S3. The difference spectrum to be used as a spectrum of Lys in solution

To show that the usage of the difference spectrum as Lys spectrum in solution is approved, in Figure S3, *a* and *b,* the difference spectrum is presented in comparison with the spectrum of Lys adsorbed to the blank crystal in an area-normalized form, for p and s polarizations. Figure S3, *c* compares the difference spectrum for both polarizations.

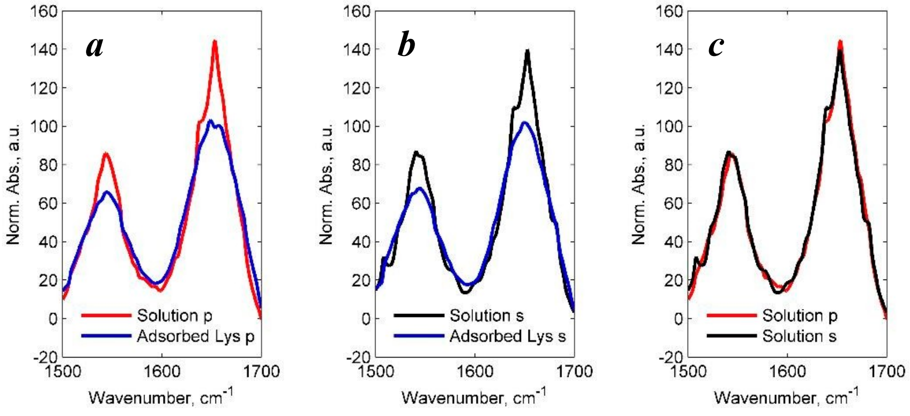

Figure S3. Comparison of the area-normalized difference spectrum with the area-normalized spectrum of the protein adsorbed to the blank crystal for the case of p (*a*) and s (*b*) polarization. (*c*) Comparison of the area-normalized difference spectrum in case of p and s polarization.

Comparing the blue lines with the red lines in Figure S3, *a* and *b* one can see that the shape of the spectra is different. The amide bands of the difference spectrum are sharper than those in the spectrum of Lys adsorbed on the blank crystal. This difference in shape suggests that the difference spectrum mostly corresponds to Lys having a structure that differs from that of Lys adsorbed on the crystal. Importantly, from Figure S3, *c* it can be seen that there is a high degree of resemblance between the difference spectrum for the case of p and s polarization. Indeed, a possibility of a preferential orientation of protein molecules in solution phase is excluded.

Additionally, it would be interesting to check whether the difference spectrum shows a similarity to the first spectrum recorded after start of Lys delivery to the blank crystal. The first spectrum recorded after start of Lys supply should be close to the spectrum of Lys in solution. Indeed, one may assume that at the very beginning protein does not have a sufficient time to reach the surface, adsorb and adopt to another conformational state, and a complete coverage of the crystal by the protein is not achieved. In Figure S4 the area-normalized difference spectrum (spectra in red and black) is compared to the area-normalized spectrum just after the start of the protein supply (spectra in blue).

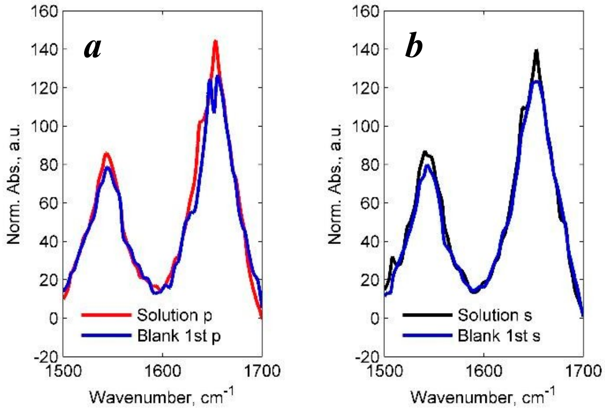

Figure S4. The area-normalized difference spectrum compared to the area-normalized spectrum recorded just after start of Lys delivery to the blank crystal for the case of p (*a*) and s (*b*) polarization.

From Figure S4, *a* and *b* it can be seen, that the shape of the presented spectra is similar confirming that corresponding secondary structure is similar as well. This is an additional indication that the difference spectrum is close to the protein spectrum in solution.

Section S4. Spectrum of Lys released form the PEM

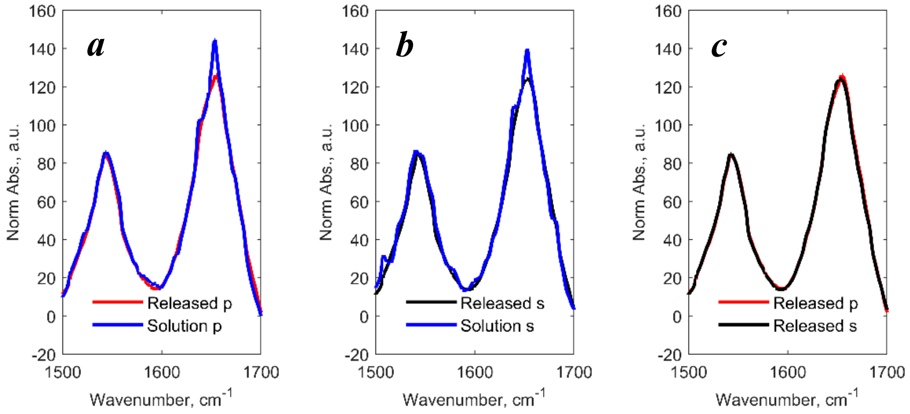

Figure S5. The area-normalized spectrum of Lys released form the PEM compared to the are-normalized spectrum of Lys in solution.

Section S5.

In Section 3.3.2.3 (see the main text) orientation of Lys adsorbed to the PEM was analyzed based on the difference spectrum obtained as a difference between the area-normalized spectra of Lys recorded with p and s polarizations. As suggested in te main text, bands observed at both 1550 cm^{-1} and 1631 cm^{-1} can be assigned to beta sheet structures [34]. The fact that this structure element (the beta sheet) is differently expressed for both polarizations in amide I and II bands can be explained in the following way. In the beta strands composing the beta sheets the transition dipole moment (TDM) that gives rise to the component at 1631 cm^{-1} (high frequency mode) has an orientation perpendicular to the TDM of the component at 1550 cm^{-1} (low frequency mode) [36,37]. Apparently, in our case the beta strand axis is oriented preferentially perpendicular to the surface of the PEM. In case of such an orientation:

- The vertical (s) polarization is highly sensitive for the detection of the beta sheets in the amide I region. The TDM of the low frequency mode is perpendicular to the vertical polarization and is almost vanished.
- In turn, the parallel polarization (p) is more sensitive for the detection of the beta sheets in the amide II region. The TDM of the high frequency mode is perpendicular to the parallel polarization and is almost vanished.

One distinguishes between parallel and anti-parallel beta sheets depending on the orientation of the beta strands within the beta sheet structure. Both these beta sheet conformations have characteristic absorbance bands in amide I as well as amide II regions. Absorbance at around 1630 cm^{-1} (amide I) is characteristic for both these beta sheet conformations [45]. Additionally, anti-parallel beta-sheets are usually characterized by a presence of a peak around 1695 cm^{-1}. From Figure 6 no increase of absorbance in the region around 1695 cm^{-1} can be concluded. This is an indication that changes observed at 1630 and 1550 cm^{-1} are due to formation/increase in amount of parallel beta sheet structures [34,35]. Formation of parallel beta sheet structures serves as a sign for formation of amyloid fibrils [34,46]. Among other reasons, the latter can be caused by temperature increase [46].

Section S6. Dichroic spectrum of Lys adsorbed to the PEM

In Section 3.3.2.3 (see the main text) orientation of Lys adsorbed to the PEM was analyzed based on the difference spectrum obtained as a difference between the area-normalized spectra of Lys recorded with p and s polarizations (Figure S6, the spectrum in green, see also Figure 7 in the main text). It is worth noting that a similar (dichroic) spectrum can be obtained from the initial, not area-normalized spectra. For this a difference of the initial spectra recorded with parallel (p) and perpendicular (s) polarizations should be calculated (Figure S6, the spectrum in blue) [15]. Importantly, the perpendicular spectrum is multiplied by a certain factor R^{iso} (a dichroic ratio for an isotropic sample) before subtraction to take into account the differences in the relative power of the evanescent fields (see Eq. (5) and (6) in the main text). For our experimental conditions R^{iso} equals 1.93 [39].

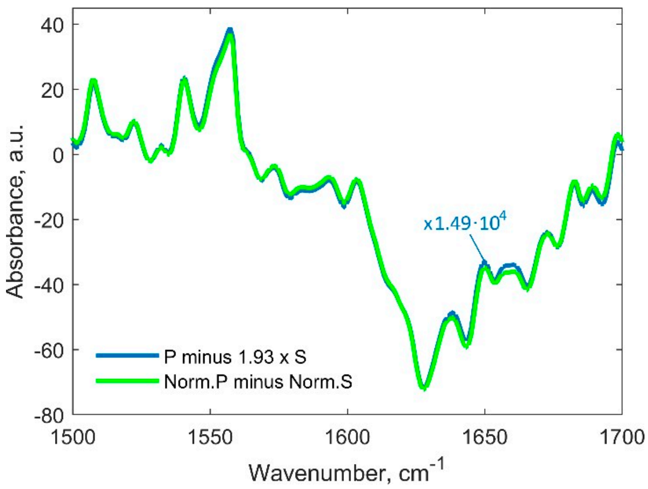

Figure S6. Dichroic spectrum of Lys adsorbed to the PEM obtained as p- minus s-spectrum after multiplication of the s-spectrum by 1.93.

One may note a strong resemblance between the spectra presented in Figure S6. This observation validates the area-normalization approach implemented in the present work for comparison of the spectra.

References

15. Bechinger, B.; Ruysschaert, J.-M.; Goormaghtigh, E. Membrane Helix Orientation from Linear Dichroism of Infrared Attenuated Total Reflection Spectra. *Biophysical Journal* **1999**, *76*, 552–563, doi:10.1016/S0006-3495(99)77223-1.
16. Keller, J. Wechselwirkungen des antimikrobiellen Peptids NK-2 mit Lipid Mono- und Bischichten. Diplom; Universität Potsdam, Potsdam, 2012.
19. *Internal reflection spectroscopy*: *Theory and applications;* Mirabella, F.M., Ed.; Dekker: New York, 1993, ISBN 0-8247-8730-7.
20. Goldberg, M.E.; Chaffotte, A.F. Undistorted structural analysis of soluble proteins by attenuated total reflectance infrared spectroscopy. *Protein Sci.* **2005**, *14*, 2781–2792, doi:10.1110/ps.051678205.
21. Sachdeva, A.; Cai, S. Structural differences of proteins between solution state and solid state probed by attenuated total reflection Fourier transform infrared spectroscopy. *Appl. Spectrosc.* **2009**, *63*, 458–464, doi:10.1366/000370209787944316.
34. Sarroukh, R.; Goormaghtigh, E.; Ruysschaert, J.-M.; Raussens, V. ATR-FTIR: a "rejuvenated" tool to investigate amyloid proteins. *Biochim. Biophys. Acta* **2013**, *1828*, 2328–2338, doi:10.1016/j.bbamem.2013.04.012.
35. Gustot, A.; Raussens, V.; Dehousse, M.; Dumoulin, M.; Bryant, C.E.; Ruysschaert, J.-M.; Lonez, C. Activation of innate immunity by lysozyme fibrils is critically dependent on cross-β sheet structure. *Cell. Mol. Life Sci.* **2013**, *70*, 2999–3012, doi:10.1007/s00018-012-1245-5.
36. Smeyers, M.; Léonetti, M.; Goormaghtigh, E.; Homblé, F. Structure and function of plant membrane ion channels reconstituted in planar lipid bilayers. In *Planar lipid bilayers (BLMs) and their applications*; Tien, H.T., Ottova-Leitmannova, A., Eds.; Elsevier: Amsterdam, London, 2003; pp 449–478, ISBN 978-0-444-50940-6.
37. Marsh, D. Dichroic ratios in polarized Fourier transform infrared for nonaxial symmetry of beta-sheet structures. *Biophysical Journal* **1997**, *72*, 2710–2718, doi:10.1016/S0006-3495(97)78914-8.
39. Dave, N.; Lórenz-Fonfría, V.A.; Leblanc, G.; Padrós, E. FTIR spectroscopy of secondary-structure reorientation of melibiose permease modulated by substrate binding. *Biophysical Journal* **2008**, *94*, 3659–3670, doi:10.1529/biophysj.107.115550.
40. Jackson, M.; Mantsch, H.H. The use and misuse of FTIR spectroscopy in the determination of protein structure. *Crit. Rev. Biochem. Mol. Biol.* **1995**, *30*, 95–120, doi:10.3109/10409239509085140.
44. Tamm, L.K.; Tatulian, S.A. Infrared spectroscopy of proteins and peptides in lipid bilayers. *Quart. Rev. Biophys.* **1997**, *30*, 365–429, doi:10.1017/s0033583597003375.
45. Miyazawa, T.; Blout, E.R. The Infrared Spectra of Polypeptides in Various Conformations: Amide I and II Bands 1. *J. Am. Chem. Soc.* **1961**, *83*, 712–719, doi:10.1021/ja01464a042.
46. Zou, Y.; Li, Y.; Hao, W.; Hu, X.; Ma, G. Parallel β-sheet fibril and antiparallel β-sheet oligomer: new insights into amyloid formation of hen egg white lysozyme under heat and acidic condition from FTIR spectroscopy. *J. Phys. Chem. B* **2013**, *117*, 4003–4013, doi:10.1021/jp4003559.
